# When risk becomes illness: The personal and social consequences of cervical intraepithelial neoplasia medical surveillance

**DOI:** 10.1371/journal.pone.0226261

**Published:** 2019-12-16

**Authors:** Carla Freijomil-Vázquez, Denise Gastaldo, Carmen Coronado, María-Jesús Movilla-Fernández

**Affiliations:** 1 Facultade de Enfermaría e Podoloxía, Universidade da Coruña, Ferrol, Spain; 2 Laboratorio de Investigación Cualitativa en Ciencias da Saúde (CCSS), Grupo de Investigación Cardiovascular (GRINCAR), Universidade da Coruña, Ferrol, Spain; 3 Bloomberg Faculty of Nursing, University of Toronto, Toronto, Canada; 4 Centre for Critical Qualitative Health Research, University of Toronto, Toronto, Canada; Universita degli Studi dell'Insubria, ITALY

## Abstract

**Background:**

After the early detection of cervical intraepithelial neoplasia (CIN), medical surveillance of the precancerous lesions is carried out to control risk factors to avoid the development of cervical cancer.

**Objective:**

To explore the effects of medical surveillance on the personal and social lives of women undergoing CIN follow-up and treatment.

**Methodology:**

A generic qualitative study using a poststructuralist perspective of risk management was carried out in a gynecology clinic in a public hospital of the Galician Health Care System (Spain). Participants were selected through purposive sampling. The sample consisted of 21 women with a confirmed diagnosis of CIN. Semistructured interviews were recorded and transcribed, and a thematic analysis was carried out, including researcher triangulation to verify the results of the analysis.

**Findings:**

Two main themes emerged from the participants’ experiences: CIN medical surveillance encounters and risk management strategies are shaped by the biomedical discourse, and the effects of “risk treatment” for patients include (a) profound changes expected of patients, (b) increased patient risk management, and (c) resistance to risk management. While doctors’ surveillance aimed to prevent the development of cervical cancer, women felt they were sick because they had to follow strict recommendations over an unspecified period of time and live with the possibility of a life-threatening disease. Clinical risk management resulted in the medicalization of women’s personal and social lives and produced great uncertainty.

**Conclusions:**

This study is the first to conceptualize CIN medical surveillance as an illness experience for patients. It also problematizes the effects of preventative practices in women’s lives. Patients deal with great uncertainty, as CIN medical surveillance performed by gynecologists simultaneously trivializes the changes expected of patients and underestimates the effects of medical recommendations on patients’ personal wellbeing and social relations.

## Introduction

Human papillomavirus (HPV) infection is one of the most common sexually transmitted infections worldwide [1] and is the cause of anogenital (cervix, anus, vulva, vagina and penis) and head and neck (oral cavity, oropharynx, other pharynx and larynx) cancers [2]. Cervical cancer is the second most common type of cancer among women living in less developed regions of the world, where there were an estimated 570,000 new cases in 2018 (84% of new global cases) [1]. In Spain, the Information Centre on HPV and Cancer estimated that 2,511 new cases of cervical cancer are diagnosed each year and that 848 deaths occur annually due to this disease, being the 10th most frequent cancer in women [3].

Two main strategies have been proposed to prevent cervical cancer: vaccination against HPV and early detection of cervical cancer precursor lesions, called cervical intraepithelial neoplasia (CIN), through cervical cancer screening [1]. In Spain, the National Health Care System is publicly funded and offers free vaccination against HPV for 12-year-old girls [4] and free cervical cancer screening (a pap smear performed every 3 years) for women aged 21 to 65 who have engaged in sexual intercourse [5]. Cytological screening is performed in primary care, and in cases of abnormal results, the patient is referred to specialized care for medical surveillance (known as follow-up) and treatment [6].

When CIN is detected in a pap smear, medical surveillance and potential treatment is performed according to the severity of the lesion (CIN 1, 2 or 3) and the individual situation of each patient (such as age, pathologies and parity). In most cases, low-grade lesions (CIN 1) are subject to cytological follow-up every 6–12 months [7] because they often (75%) are transitory and spontaneously return to normal without leaving any type of injury [8]. However, high-grade lesions (CIN 2–3) may require treatment (cryotherapy, loop electrosurgical excision procedure (LEEP) or cold knife conization (CKC) and medical follow-up [9].

The recommendations that gynecologists give women during medical follow-up to prevent CIN from evolving into cervical cancer are related to behavioral risk factors. These recommendations differ in some countries, given the varied results in the biomedical literature. Specifically, in Spain, women are strongly recommended to stop smoking since smoking has been identified as a major risk factor [10]. In addition, women are told to use condoms during sexual intercourse. Although condom use does not completely prevent the transmission of HPV, some studies have suggested that if used correctly and consistently, condom use reduces the risk of lesion progression and supports lesion regression [11]. Finally, women with CIN are encouraged to be vaccinated against HPV. While vaccines against HPV have not shown therapeutic benefit in patients with CIN, they have shown efficacy in preventing reactivation or reinfection by the types of HPV included in the vaccines [12].

While most international publications have focused on the biomedical aspects of CIN that support its clinical management, a few studies have explored the experiences of women with CIN and the consequences of medical surveillance and treatment. Most women with CIN lack knowledge about their conditions [13–15] and describe poor professional-patient communication [15–17], which hinders patients’ ability to take responsibility for self-care [14, 18], reduces adherence to medical follow-up [18] and might cause negative psychological effects [13, 19], such as anxiety [14, 16] and fear of cancer [14, 17]. The close link between CIN and sexuality, fidelity, and religious beliefs, as well as the connotation of promiscuity, causes women to experience stigmatization and self-blame [20, 21]. In some cases, women avoid talking about CIN to preserve their relationships with their families or partners [20], which limits their ability to seek support and causes them to distance themselves from their social environments [22]. Several studies have described that different priorities in patients’ lives, such as having children and maintaining the bond of marriage and sexuality, are threatened when they receive the diagnosis [17, 22, 23]. Although these women understand that CIN has not been the result of infidelity, distrust persists, causing disagreements, reproach and, in some cases, the breakup of a couple [17]. However, in Spain, there are no studies about women’s CIN follow-up experiences.

To further explore this topic, we utilize a poststructuralist perspective of risk management to understand how knowledge and power are constitutive elements of medical surveillance [24]. According to Foucault, medicine has become an increasingly powerful institution in Western countries as promoting biological life has become a political phenomenon supported by our current shared desire for healthy, long lives–what Foucault called biopower [25, 26]. In this context, most patients and citizens accept the authority of biomedicine and the power of physicians and other health care providers to prescribe behaviors and tell patients how to live (e.g., diet, exercise, and sexual practices). Patients volunteer their bodies to be examined through invasive and sometimes embarrassing tests as in no other form of social encounter because they share the dominant view that such scrutiny is beneficial [27]. In the modern doctor-patient relationship, power is exercised through an expectation of compliance to prescribed practices, even when the patient cannot understand the rationale that justifies them.

With the expansion of medicine into a large complex of specialties and realms of practice, contemporary biopolitics has been defined by health interventions that are no longer intended to only treat disease but also to address the "treatment of risks". Risk management, such as the risk profile, is closely related to interventions that establish who is presently considered “high risk” to establish ways of living that support a more desirable and less ill future. Western liberal democracies have committed to the optimization of lifestyles, health and quality of life to the point that engagement with such rationality is almost mandatory [28]. It has become expected that individuals take responsibility for protecting themselves and their families from multiple forms of risk. Therefore, the medical encounter is dominated by the expectation that a person who is considered "at risk" will take action to avoid risks [29].

Women, in particular, are expected to engage with preventative measures, not only for themselves but also for others, as there is a social expectation in patriarchal societies that women will be the main caregivers for children, family members and their partners [30]. However, in the context of sexually transmitted infections, women’s morality comes into question, and how they are perceived by professionals may interfere with how they experience the medical encounter. The dominant discourse sets expectations for women to be sexually inhibited, and being seen as promiscuous would be offensive for many [31]. The conflation of these two discourses shapes CIN clinical encounters in relation to the ideas that are taken for granted, the questions that are asked, and the advice that is provided.

Internationally, few studies have focused on the study of risk management through medical surveillance (follow-up) of HPV infection. Most studies have focused on vaccination against HPV in Canada [32–35], the United States [36] and Australia [37]. Other studies explored how individual women deal with and react to information on cervical cancer risk in the context of cervical cancer screening in United Kingdom [38] and Australia's gendered approach to health care rights surrounding pap smear registration and testing [39]. Only one Canadian study [40] juxtaposed women’s experiences of precancer diagnosis and treatment with the decision to have the HPV vaccine, concluding that these women feel neither well nor sick, as they face a chronic infection that is very difficult to manage and that makes them feel at risk of developing cervical cancer.

Given the limited research on the subject, the aim of this study is to explore the effects of medical surveillance practices, which we call “treatment of risk”, on the social and personal lives of women undergoing CIN medical follow-up and treatment.

## Methods

A generic qualitative study was carried out [41]. The study received approval from the Autonomous Committee of Research Ethics of Galicia (registration code: 2015/230), and access to the setting was authorized by hospital management.

The study was conducted in a gynecology clinic of a public hospital in the Galician Health Care System in northwestern Spain. Recruitment was carried out from October to December 2015. Purposive sampling was used to recruit participants according to the study objectives [42]. Participants were selected based on the following inclusion criteria: Spanish speaking women between 21 and 65 years of age with a CIN diagnosis of any degree. Women with a diagnosis of cervical cancer or with physical or mental comorbidities that interfered with the description of the follow-up experience were excluded.

The first author (CFV) accompanied the gynecology team during their consultations and personally explained the study to potential participants after their appointments. At that time, she also invited women to participate in the research and asked for a phone number. Two days after recruiting each woman, the researcher called her to set up an appointment for an interview in a hospital room reserved exclusively for this study. Thirty-one women were contacted throughout the recruitment process, ten of whom decided not to participate. Five of them mentioned personal reasons when they were called, and another five women did not attend the appointment without prior notice or justification.

At the interviews, the researcher explained in detail her academic credentials (BSc(N), MSc), the aims of the study, the reasons for carrying out the study and what participation consisted of. The participants were given an information sheet and the informed consent form, which was explained and signed before the interview.

The interviewer conducted individual semistructured interviews using a guide based on a literature review and the advice of three experts, two in qualitative methodology (MJMF, CC) and one in HPV infection. By combining the results of the literature review and the advice of the HPV infection expert, we were able to develop comprehensive, in-depth questions to address the follow-up experiences of women with CIN. In addition, the review of the questions by experts in qualitative research allowed us to ensure that the questions were methodologically sound (see S1 and S2 Tables—Semistructured interview guide).

Data collection ended with the 21^st^ interview, when data saturation [43] was reached. In Table 1, the sociodemographic characteristics of the sample are described. The age range of the participants in the final sample was 21 to 52 years (mean age: 34 years). At the time of data collection, 15 participants were in risk management follow-up, and six were in follow-up after conization; their follow-up time ranged from one to ten years (average years in medical follow-up: 3 years).

**Table 1:** Sociodemographic factors.

Participant	Academic level	Marital status	No. of children	Type of diagnosis	Year of diagnosis
**I-1**	Graduate degree	Single (with partner)	0	CIN 1	2013
**I-2**	Compulsory secondary education	Single (with partner)	0	CIN 2	2015
**I-3**	Higher vocational training	Single (without partner)	0	CIN 1	2008
**I-4**	Graduate degree	Married	2	CIN 1	2015
**I-5**	Graduate degree	Single (with partner)	0	CIN 3	2015
**I-6**	Baccalaureate degree	Single (with partner)	0	CIN 3	2013
**I-7**	Associate degree	Single (with partner)	0	CIN 1	2012
**I-8**	Graduate degree	Single (with partner)	0	CIN 1	2014
**I-9**	Compulsory secondary education	Married	2	CIN 1	2014
**I-10**	Graduate degree	Married	1	CIN 3	2010
**I-11**	Higher vocational training	Separated (with partner)	1	CIN 2	2012
**I-12**	Graduate degree	Single (with partner)	0	CIN 2	2014
**I-13**	Higher vocational training	Married	1	CIN 1	2015
**I-14**	Graduate degree	Married	1	CIN 1	2010
**I-15**	Higher vocational training	Single (without partner)	0	CIN 1	2012
**I-16**	Associate degree	Single (with partner)	0	CIN 1	2007
**I-17**	Graduate degree	Married	0	CIN 1	2013
**I-18**	Graduate degree	Married	0	CIN 1	2010
**I-19**	Associate degree	Divorced (with partner)	1	CIN 1	2015
**I-20**	Baccalaureate degree	Married	2	CIN 3	2005
**I-21**	Higher vocational training	Single (with partner)	0	CIN 1	2014

The interviews were conducted from October to December 2015. The majority of the interviews lasted approximately 40 minutes, but a few participants had particularly short interviews (two interviews lasted less than 15 minutes). We assume these participants were trying to help but did not understand the format of the research interview or wanted to avoid difficult subjects. Other interviews were closer to an hour, suggesting the participants’ desires to contribute through detailed descriptions of their experiences. The interviewer had never worked in the research setting and had no prior relationships with the participants. This allowed participants to talk freely about their perceptions during the interviews without feeling that their participation would interfere with health care provision. Due to the information needs detected, after the interview, the researcher responded to questions raised by the participants that generated distress or unnecessary concern.

The interviews were audio recorded and transcribed, and the recordings were destroyed after the verification of the transcription accuracy. Field notes were written after each interview and integrated into the transcripts. We conducted a thematic analysis with inductive and deductive elements, identifying units of meaning and assigning codes, which were grouped into categories [44, 45]. The summary of the analysis is shown in Table 2. Throughout the process, analytical memos were developed, and the ATLAS.ti computer program (version 7.5.10) was used to manage the data analysis.

**Table 2:** Summary of the analysis.

**Theme 1: Cervical intraepithelial neoplasia medical surveillance encounters and risk management strategies are shaped by the biomedical discourse**	
**Subcategories:**	
Cervical intraepithelial neoplasia medical monitoring to prevent cancer New disease status for patients	
**Theme 2: Effects of “risk treatment” for patients**	
**Subcategories:**	**Macrocodes and codes:**
Profound changes expected of patients	• Uncertainty in the health care process • Effects on pregnancy planning • Effects on sexuality
Increased patient risk management	Managing self-risk • Need for more follow-up consultations • Need to undergo conization • Need for private gynecological assistance • Need to check the oral and anal cavities • Avoidance of sexual relations with the partner • Good nutrition for cancer prevention • Cancer genetics as a risk factor • Cancer as a prevalent disease Managing others’ risk • Need for medical assistance for men • Awareness of other women
Resistance to risk management	• Lack of information to make decisions • Effects on sexuality • Non-use of condoms • Not receiving the vaccine • Not giving up tobacco • Desire to forget the illness

To ensure trustworthiness [41, 46], researcher triangulation was carried out among the three researchers (CFV, MJMF and DG) who read and analyzed the transcripts, and the final categories were agreed upon by the entire team. Any doubts were clarified during the interviews since the interviewer only met with each participant once. Strategies to increase the rigor of the study included the use of reflexivity and positionality (e.g., discussing how our professional education influenced how we understood participants’ perspectives) and the recording of all key methodological decisions throughout the study to be able to report them accurately.

## Results

Two main themes were identified through our analysis: CIN medical surveillance encounters and risk management strategies are shaped by the biomedical discourse, and the effects of “risk treatment” for patients include (a) profound changes expected of patients, (b) increased patient risk management, and (c) resistance to risk management.

Together, these two themes reveal that CIN surveillance is problematic from patients’ perspectives and has consequences for their lives, but patients react in different ways–what we have conceptualized as the effects of medical surveillance. We discuss three types of effects, similar to a typology, but many participants described experiencing mixed effects on their personal and social lives. While we noticed that participants tended to comply with the medical discourse, they also went beyond what their health care providers asked, increasing their levels of personal risk management and/or resisting risk management recommendations.

### CIN medical surveillance encounters and risk management strategies are shaped by the biomedical discourse

We start by offering context to how the medical surveillance process typically occurred according to the participants and documents from the clinic. A clear biomedical rationale guided the entire process. At the first consultation, gynecologists explained to women with CIN what the HPV infection was and how to prevent cervical cancer. In the brochure that was given to patients, the importance of the follow-up and/or treatment of their precancerous lesions was clearly described:

*“When the infection in women persists over time*, *usually years*, *and certain factors coexist*, *with the main one being smoking*, *there will be a greater probability of a cervical lesion occurring (CIN)*, *which if left untreated*, *may end up as cervical cancer*. *The infection is not treated since it resolves itself in most cases*, *but the long-term consequences of the HPV infection are treated”* (Field notes: Nov 2015). The brochure also stated that the treatment would be individualized for each woman’s case: *“In some cases*, *observation and follow-up are sufficient [every 4*, *6*, *12*, *24 months or 3 years*, *depending on the situation of each patient]*, *and others will need treatment (cryocoagulation*, *laser vaporization or conization) with follow-ups”* (Field notes: Nov 2015).

Due to the lack of a treatment for HPV infection, the participants’ gynecologists made a series of recommendations aimed at controlling the risk factors to avoid a future cancer: to quit smoking and to use condoms consistently and appropriately. In addition, the patients were informed that they should receive the HPV vaccine.

Compliance was positioned as the basis for risk management. For women with CIN, a nonchalant approach was used, including reassurance that their lesions (CIN) were only in a precancerous stage and that medical surveillance and compliance with the recommendations were very important to avoid developing cancer in the future. This information was conveyed to women during consultations, as explained by one participant:

*“The exact words from him [the gynecologist]*: *‘It’s not cancer today*, *but if we don’t treat it*, *it can be one day’ (…) I was not expecting this*, *and I fell apart (…) He told me*, *‘Don’t worry…’ (…) He let me know that it wasn’t that serious*. *I remember telling him*, *‘But*, *do I have to worry about it*?*’ And he replied*, *‘Not for now*, *but if we don’t treat it*, *you might have to one day’*. *So*, *his words have stuck in my head ever since*.*”* I-10

While the participants’ gynecologists were managing risk to prevent a future health problem, the women felt like they were sick, in treatment, and under surveillance for a potential new disease that was going to be managed over time. Being diagnosed with CIN meant that patients had to live with uncertainty for a long time, if not forever, facing the possibility of developing cervical cancer. As one participant described,

*“I have something that can cause cancer (…) When I left [his office]*, *I wasn’t angry or confused*, *I was just sad*. *Not because I may have cancer one day*, *not even because I might die of this (…) it was more in the sense that I was 22 at that time… and to be faced with such a big thing at such a young age*, *you know*? *The infection… something like this*, *it’s going to be part of me for years*.*”* I-18

### Effects of “risk treatment” for patients

Women with CIN reacted to the biomedical discourse on risk management in different ways. Most women complied with the medical recommendations, while others increased their personal risk management strategies beyond what was proposed; some also resisted the proposed risk management strategies, questioning several elements of what they were told to do.

#### (a) Profound changes expected of patients

Although the medical encounter is based on patient compliance with disease treatment, the management of risk factors adds a degree of complexity that makes it difficult for patients to comply, even when they want to. In the case of CIN, risk management encompasses key elements of life and can have significant, enduring consequences for patients’ lives (e.g., having children). The participants in our study who wanted to fully comply with the provided recommendations experienced periods of great uncertainty. They had to wait for long periods of time between consultations, being unsure about recommendations that impacted their sexuality and potential motherhood. One participant explained this uncertainty as follows:

*“You have to change lots of things all of a sudden (…) Every new appointment with the doctor was something new to us; you were being told what you could and couldn’t do*. *So… we had the usual anxiety perhaps*, *you know*? *We went from… feeling calm to this new unexpected situation*. *You see*, *it was the usual thing*, *living your sexuality as a totally normal thing and*, *suddenly*, *you are told all these new rules like*, *‘Remember to use condoms; forget about oral sex for now’ (…)*.*”* I-1

The prescription of indefinite condom use greatly interfered with pregnancy planning for our participants, leading to conflicts for some couples. When the participants asked their gynecologists when they could become parents, they did not receive specific answers, which led to continuous uncertainty, as the following quotes reflect:

*“It was a disappointment not being able to have a child*, *as we had to wait and see how the condition would progress (…)*. *We had been together [as a couple] for 8 years*, *and we were getting old*. *I remember my husband asking in one of the sessions*, *‘So*, *maybe we can’t have children; is that right*?*’ And the gynecologist replied*, *‘What do you prefer*, *your wife’s health or having a child*?*’ So*, *my husband said to her*, *‘Of course*, *I choose my wife’s health’*. *We wondered if we could ever have them [children]… either now or in the future*.*”* I-10 (In this case, the patient’s partner decided to attend the appointment with her, but the health care system protocol does not include any kind of assistance for men.)

*“So*, *he [the participant’s partner] started saying something like*, *‘Damn it…what about having babies*?*’ ‘No*, *we can’t have children*. *Not until a year or two have passed’ [the participant replied]*. *‘Damn it… a year or two… but what if after a year or two the result [from the pap smear] comes out wrong again*?*’ [her partner asked]*. *‘We’ll never have children then*!*’ [she replied]*. *Of course*, *all of this makes you feel terribly sad*.*”* I-21

There was tension between the logic used by the gynecologists (risk management) and the patients (being sick and having their lives altered by this condition). The participants knew that they did not have cancer in the present, but they had to follow strict recommendations for an extended period of time, based on the logic of surveillance and risk management, which was experienced as being under treatment.

The participants’ uncertainty regarding the prognosis of their condition was experienced like an illness:

*“They do check-ups to make sure it [CIN] is not progressing and that everything is fine*, *but… am I going to be like that my whole life*? *(…) I was feeling anxious because the doctor said it was serious (…) I went home thinking… I am sick…”* I-18

Within this logic of prevention and risk management, some participants went far beyond what was recommended, aiming to prevent the transmission of HPV during sexual intercourse and the development of CIN into cancer.

#### (b) Increased patient risk management

Feeling at risk of developing cervical cancer made some participants want to control most aspects of their lives to address uncertainty. Given that they needed to be under constant medical surveillance, the participants expected the same treatment for their partners, avoided sexual intercourse to prevent HPV reinfection and sought out other protective factors (e.g., a healthy diet) or additional information about cancer-related issues (e.g., reasons for a high prevalence of cancer or a family history of cancer). In addition, they raised awareness regarding HPV infection so that close relatives and friends would not suffer from the same condition.

The participants proposed that in the absence of a treatment to cure “the disease”, they should increase the number of follow-up visits, especially in cases of a CIN 1 diagnosis.

*“If it were up to me*, *I would come [to an appointment] every month*. *It would be ideal for me*. *The thing is that I’m afraid that with time*, *this can turn into something more serious [cancer]*. *I would like to get checked more often (…) How come there is no pill to take for when you are in the first stage [CIN 1]*? *Or something to treat this so that it doesn’t develop any further into the second stage [CIN 2]*?*”* I-11

Some participants wanted to undergo a conization, even though it was not indicated in their cases, because they thought that it would "cure" CIN and prevent the development of cancer.

*“I was given the results… I had come with my mother… and I told her*, *‘I really hope I’m told I am having this [CIN] removed as soon as possible*. *I want to have a conization done right away’*.*”* I-18

As users of the National Health Care System in Spain, the participants attended the necessary consultations to undergo the required tests (i.e., pap smears, biopsies and colposcopies) and obtained the test results. However, some women chose to seek “treatment” with a private gynecologist as well to feel safer while still under medical surveillance in the National Health Care System. Private care allowed follow-up consultations on demand, including tests for their partners and of other areas of the body, such as the oral and anal regions. Such proactive approaches reveal how much some participants engaged in self-surveillance and extended surveillance to others. This represents a class-based strategy since women who did not have economic means could not access the private system.

*“When I went into the doctor’s office [in the National Health Care System]*, *every aspect seemed to be directed towards me*. *I found it really strange that–given the nature of this pathology–I wasn’t asked any questions about my partner*. *I was expecting to hear something like*, *‘What kind of sexual behavior did your partner have*? *How long have you been with him*? *How is he*? *Have you seen any little warts on him*?*’ I believe this pathology affects us both*. *I went to many private doctors with my partner*, *so we were both checked*. *We also had our mouths checked because I was afraid about all that too*.*”* I-1

In addition to extending medical surveillance to their partners, some participants performed actions that were in their power to manage risk daily. The difficulty of managing reinfection risk during intercourse affected couples’ sexuality. The participants took all precautions to avoid the transmission of HPV during sexual intercourse, even avoiding it since abstinence is the only fully safe measure. This everyday risk management performed by women living with CIN affected the sexual health of the participants and their partners.

*“It affected me because for a time*, *I didn’t want to have sex with him [her partner]*, *and I was obsessed with the idea that he was the one with the virus and I was getting infected all the time*.*”* I-3

The participants also applied general scientific ideas related to cancer prevention to their risk management strategies. For instance, given that diet influences health status, some of the participants also changed their food intake, as described by one participant:

*“I try to eat healthy*. *I don’t like fatty foods; I eat grilled vegetables … but since I found out about this [the CIN diagnostic]*, *I was like*, *‘I’m going to try to do all that I can so that this works’*. *I bought a big blender and all possible fruits*. *Vitamins*!!! *Let there be vitamins in my body in case it is worth something*.*”* I-3

The participants explained their predispositions for developing cervical cancer through epidemiological data on the high prevalence of cancer in the Western world described in the media. They also identified genetic predisposition as a risk factor.

*“There is a genetic predisposition in my family*, *and you know that sooner or later*, *it’s going to be your turn (…) My dad*, *my grandad*, *all my grandfather’s brothers died of cancer (…) Why isn’t it going to affect me as well*, *as everybody in my father’s family has died of this*? *Also*, *statistically speaking*, *there is also a chance*, *you know*? *I suppose that at any moment*, *I’m going to be told*, *‘You have this condition [cancer]*, *and we are going to treat you’*.*”* I-16

This increased risk management was not limited to participants and their partners but also was extended to their family members and friends, raising awareness about preventive activities so that they could avoid HPV infection.

Occasionally, in search of a comprehensive cancer prevention plan, the participants acted upon perceptions of risk to others. However, some of the actions they carried out were not based on scientific knowledge because they lacked information about their condition (authors, published article) [47]. For instance, one participant said,

*“I have shared the same towel with my sister*. *Also*, *our underwear is together before it gets washed… I don’t know… is it infected then*? *Can I pass this on*? *I have no idea*. *So*, *I told my sister to go to the doctor to have a pap smear done to test for HPV*. *My sister replied*, *‘How am I going to tell that to the doctor*?*’ I insisted*. *The doctor refused to do it*.*”* I-21

Despite the efforts to comply and engage with health promotion and disease prevention strategies by the majority of participants, not all participants decided to comply with the biomedical discourse of risk management. Several acts of resistance against the recommendations were also described.

#### (c) Resistance to risk management

In most cases, the logic used by the participants to not follow some recommendations was based on their lack of understanding of the purpose of the recommendation. The participants stated that they were not taken into account in decisions about their self-care; rather, they were just told what to do, which had impacts on their adherence. As the following quote explains, there was an expectation of compliance but, in some cases, an unwillingness to explain to the patients the reasoning behind the recommendations:

*“You feel exhausted because the gynecologists put some pressure on you [pressure to comply with the recommendations] (…) You feel under great pressure*. *It goes something like this [conversation]*: *‘Were you vaccinated*?*’ ‘No*, *I haven’t made up my mind yet*.*’ ‘Ah*, *it’s up to you then*.*’ I mean*, *perhaps she [the gynecologist] could have said something like*, *‘Why are you having problems making the decision*? *What kind of doubts do you have*?*’ However*, *this was not the case*.*”* I-16

Always using condoms during sex implied a change in the experience of sexuality for the participants and their partners. As the next two quotes reveal, the fact that condom use is not a fully effective strategy compromised the recommendation. In addition, the adherence to the recommendation decreased when the participants’ partners did not want to use condoms because they felt it interfered with sex and decreased sexual pleasure, which generated relationship tensions.

*“They say that even with a condom*, *contact with the skin is enough to transmit it*. *So*, *condoms are not very effective because they don’t cover the whole area [all the genitals]*.*”* I-19

*“No [I don’t use condoms]*. *I have an IUD*. *At the beginning*, *when I started going out with him*, *I would tell him to use a condom*. *As the relationship progressed*, *I got an IUD*, *and we stopped using condoms*. *Honestly*, *I think men don’t like wearing condoms; they don’t feel comfortable using them [either]”* I-19

The participants understood vaccination to be a health practice to prevent disease because they explained that they did not see a reason to be vaccinated if they had already been infected. Other critical elements identified by women regarding vaccination were the known side effects as reported in the media and the high cost of the vaccine, as described in the following quote:

*“The gynecologist recommended I get the vaccine… but this was 7 years ago*. *In that moment*, *there was a lot of debate about this issue*. *I remember seeing a girl on TV that had gotten sick after getting the vaccine*, *so I told her [the gynecologist] I wouldn’t do it*, *given that it was all fairly new and maybe the whole thing was not yet fully developed*. *I was afraid*.*”* I-3

The logic used by the participants not to stop smoking was that they needed to smoke to manage anxiety and that they could not quit at a time when they were anxious because of CIN.

*“The doctor told me*, *‘You shouldn’t smoke; better still*, *you must not*.*’ I replied*, *‘After all the things you’ve told me*, *the damage you’ve caused and how dizzy I just felt*, *I think I will smoke 3 cigarettes at once as soon as I get to my car*. *Don’t tell me not to smoke when I get to my car because in the state of nerves I’m in right now*, *I’m going to do it’*. *‘Well*, *you shouldn’t’ [the gynecologist replied]*.*”* I-2

The CIN diagnosis weighed on the daily lives of women. As a stress management strategy, they tried to "forget" their infection and lesions to be calmer, as described below:

*“It’s not like I have forgotten about it; I do try to forget*. *But*, *it’s always in my mind (…) It’s not like I didn’t think it was important at first; it’s more like I needed to reduce its level of importance*. *If I spend all day thinking that something bad is going to happen to me*, *I won’t be able to leave my house*. *So*, *this is me now; I prefer to forget about this altogether… so to speak*.*”* I-13

While all participants wanted to be healthy, their acts of resistance show that risk management spreading into many domains of life will inevitably clash with other priorities, needs and desires that constitute patients’ personal and social lives.

## Discussion

This study reveals how patients’ daily lives are affected by risk management in CIN medical surveillance. It makes an original contribution to the understanding of the effects that risk discourses have on women’s lives and to the problematization of the competing logic that patients and gynecologists employ during the medical encounter, with the former “feeling sick” and the latter “treating risk” to avoid disease.

In summary, we classified our main findings as follows: CIN surveillance encounters and risk management strategies are shaped by the biomedical discourse, and the effects of “risk treatment” for patients include (a) profound changes expected of patients, (b) increased patient risk management, and (c) resistance to risk management.

Our study points to the challenge of risk management in clinical practice with information based on epidemiological data. Such an idea was established decades ago in a classic study about benign breast lumps in the US, where Gifford [48] explored the ambiguities that clinicians and patients face in risk management. Gifford argued that doctors were increasingly forced to apply general epidemiological knowledge of risk within populations to specific individuals, knowing that the diagnosis does not predict a future disease with any certainty. However, it is surprising not to see greater engagement with the unanticipated and mainly undesirable effects that risk management has for patients in the international medical literature.

In general terms, the uncertainty present in medical surveillance results in the medicalization of women's lives [27]. For our participants, the need to follow strict recommendations over an unspecified period of time made them feel sick, especially in the context of preventing a life-threatening disease. Risk management produced uncertainty for patients because it was not understood as prevention but rather as treatment. Similar to our findings, the findings of Wyndham-West et al.’s [40] study with women with a cervical precancer diagnosis in Canada showed that the participants described feeling neither well nor sick. Studies have shown that while gynecologists’ approaches to CIN were focused on the prevention of cervical cancer [1, 5], which is very prevalent worldwide [1], they did not include other forms of HPV-produced cancer prevention (such as oral or anal cancer) or include women's partners during the follow-up process [5]. Such rationality based on prevalence created considerable uncertainty among our participants, who expected a more comprehensive approach to health care. From a patient perspective, any cancer is potentially a death sentence; it does not matter how prevalent the cancer is in the population [17, 22, 23].

Within this context, the first effect of medical surveillance we identified was the profound changes expected of women living with CIN. In short encounters over several months or years, they were told by gynecologists to change their sexual practices, quit smoking, and/or postpone or avoid having children. As described by Pessanha-Carvalho and colleagues [49], in CIN surveillance, the sexuality and fertility of women, which are crucial elements for women’s subjectivity, are threatened, and as seen in our study women’s partners’ parenthood plans are also threatened. Hernández-Corrochano [50] explained in her study with older Spanish primiparous women that there is social pressure for women to be mothers. Therefore, Spanish patriarchal culture could amplify the psychosocial impact of postponing motherhood because women with a CIN diagnosis do not know if they will be able to be mothers. In light of our results, professionals conducting CIN follow-ups should take into account women's specific concerns about fertility and pregnancy planning. As shown in cancer and fertility research [51, 52], the possibility of fertility-sparing approaches to preserve the reproductive potential of women affected by cervical cancer should be discussed with patients.

In addition, there are currently several forms of evidence supporting the use of specific biomarkers to identify early-stage cervical cancer, which would allow for a better prognosis [53, 54], diminishing patients’ uncertainty. However, until this option is implemented in clinical settings, it is important to understand the effects of uncertainty on patients’ wellbeing.

As Lupton [55] described, doctors have been considered to be dominating and coercive in their uses of biomedical reasoning, either consciously or unconsciously repressing patients’ autonomy. According to our participants, they and their partners were occasionally reproached if they questioned the information provided or did not fully comply with recommendations. During consultations, power relations based on knowledge produced dynamics that sometimes were not in the best interest of all parties. Lupton [55] noted that in the context of health care, it is incumbent upon both doctors and nurses to objectify patients at some level, and patients frequently accept and expect that in their search for appropriate medical care. However, we believe that creating distress and relationship tensions and not supporting patients’ understandings of their own condition are effects that should be challenged.

The second effect described by the participants was increased risk self-management. Participants engaged with scientific information and information in the media in addition to their own health beliefs to expand their repertory of disease prevention strategies in the hopes of “curing” the HPV infection and avoiding HPV reinfection and CIN progression. This form of self-care and personal responsibility is an effect that has been well described in Foucauldian studies. For example, in a study of women with a genetic risk for breast cancer, Polzer [56] described that while women could not control breast cancer or prevent the disease completely, they felt that, through their participation in risk management activities (e.g., diet, exercise), they could exercise control over certain aspects of their health in general–similar to our participants. We consider that constantly engaging in health promotion strategies and seeking additional medical surveillance (e.g., private gynecological consultations, partner screening, check-ups to detect oral or anal cancer) should not be seen as benign strategies but rather as practices that have emotional consequences, as our results show.

Armstrong, in her study of cervical cancer screening [38], suggested that the risk factors of cervical cancer are mainly described to be related to women’s individual behavior (i.e., smoking and sexual behavior), which makes them believe that they can change their behavior responsibly to avoid future illness. As Gastaldo [26] proposed, this sense of control over health-promoting behavior to prevent or face disease is a dominant discourse in the health sciences and an element that promotes personal responsibility above other understandings. Most of the time, CIN disappears on its own [8], but rather than emphasizing this fact, health care professionals instead highlight patients’ individual management of risk without an examination of the emotional, personal and social consequences of this process.

In addition to feeling responsible for managing their own risk, our participants wanted to manage the risk of their loved ones, preventing them from acquiring HPV. Some of our participants even avoided sexual intercourse to manage their own risk and their partners’ risk; however, Wyndham-West et al. [40] noted that, even though their study participants recognized that only by avoiding sexual intercourse would they be fully protected against cervical cancer, they did not consider sexual abstinence for an undetermined number of years to be a viable option.

The participants in our study also wanted to increase the awareness of HPV infection among the women close to them. The findings on the role assumed by our participants as health promoters and caregivers are supported by other Spanish authors [30, 57], revealing there is a social expectation that women are responsible for caring for and protecting the health of family members, partners and people closely related to them.

The third effect, resistance to the biomedical discourse, was observed for patients who rejected condom use, kept smoking, did not receive the HPV vaccine or actively tried to forget that “they were sick/at risk” to feel better. A few studies have described why women with CIN resist medical advice. Researchers Markovic-Denic et al. [14] and Fish et al. [18] proposed that a lack of adherence to recommendations is due to women’s insufficient knowledge to make decisions about their self-care. Supporting the assertion of these authors, some of our participants decided not to comply with recommendations because health professionals did not explain the reasons or benefits of carrying them out. However, a novel finding of our study was that not all women decided to resist medical advice due to a lack of knowledge. Instead, some women with CIN decided to resist medical recommendations because complying with them had major social and personal consequences. Our study revealed that women made decisions as individuals, members of a couple, family members, and society members. They were interested in the prevention of cancer for everyone involved in their lives but also in other aspects of their lives (e.g., parenthood), which could lead to resistance to some recommendations.

Accordingly, some of our participants were reluctant to be vaccinated against HPV. Like the participants in Wyndham-West et al.’s study [40], the participants in our study believed that the HPV vaccine might not be effective since they already had the infection and knew that the vaccine was expensive. Relationship tensions due to uncertainty and the negotiation of consistent use of condoms were some of the consequences of the proposed individualized risk management strategies that disregarded the personal and social repercussions such strategies might have. International studies evaluating other groups’ reasons not to adhere to condom use have shown that conflicts could arise from couples’ condom use negotiations [58] and that men were noncompliant with condom use due to perceived reduced sexual pleasure [59]. Such conflict and men’s perceptions of reduced sexual pleasure were some one of the reasons why women in our study did not use condoms consistently. They preferred that sexual relations be satisfactory for both partners, taking into account their male partners’ perspectives on condom use. In terms of smoking and stress associated with the disease, the findings of studies of patients living with human immunodeficiency virus are consistent with our findings. Previous studies have shown that people did not stop smoking because it was a stress management strategy [60] and that patients adopted avoidant emotional coping strategies to be calm [61].

## Conclusions

In medical encounters for “treating CIN risk”, physicians used epidemiological knowledge to manage individuals’ risk, thereby creating uncertainty; however, patients saw medical surveillance as beneficial for their health. In this context, it is assumed by physicians (and society) that patients will comply responsibly with medical recommendations, even when patients do not understand the rationale that justifies them.

We argue that while physicians treat risk as disease prevention, patients think of risk as being sick. Because they are told to carry out rigorous preventative practices over a period of time and to continue to see the gynecologist, and because they sometimes receive treatment to eliminate CIN, they perceive the process as an illness. These different rationalities coexist and compete in the clinical encounter, making it a contentious space where the biomedical discourse influences patients’ perceptions and wellbeing.

Our study shows that patients deal with great uncertainty, as CIN medical surveillance performed by gynecologists simultaneously trivializes the changes expected of patients in their everyday lives and underestimates the effects of medical recommendations on patients’ personal wellbeing and social relations. Our participants reacted to the biomedical discourse on risk management in different ways, including by complying with medical recommendations, increasing their personal risk management strategies beyond what was proposed and resisting risk management strategies.

### Implications for practice, professional development and research

Gynecologists and other health care providers could provide better psychosocial support to women with CIN if they considered the potential effects of the risk management practices they recommend. Medical advice during consultations should acknowledge the specific features of patients’ psychosocial lives (women and their partners, who can be male or female and cisgender or transgender). There is a need to treat couples as well as individual women, systematically addressing their concerns to reduce uncertainty and negative personal and social impacts.

According to the results obtained in our study, to meet the needs of women diagnosed with CIN, health care providers should consider taking the following steps during medical appointments: 1) provide verbal and written information related to CIN medical follow-ups and treatment, explaining the reasons why the different clinical recommendations are given; 2) acknowledge that patients have their own rationalities and information about risk management based on social norms and social media; 3) address the concerns of women and their partners regarding sexuality (including vaginal, oral and anal sex) and reproduction; 4) explore how patients and couples engage with risk management and health promotion and the effects of such practices for themselves and others; 5) inquire about the reasons why women do not comply with clinical recommendations in a nonjudgmental manner, supporting their decision-making processes; and 6) offer health resources to help women who want to quit tobacco and be vaccinated against HPV.

Improved care will not necessarily lead to full compliance. We believe that there will always be competing discourses in clinical encounters that create resistance, as biomedicine has an individualistic, body-based approach to health, while patients are social beings living in a complex world who want to be healthy but also want to have emotional and socially meaningful lives.

In the future, to obtain more specific results, researchers should consider some of the limitations of this study. We utilized an exploratory sample, but purposively recruiting patients of different ages and educational levels could reveal more nuanced effects related to age and education. Finally, more research is needed to further investigate the ample consequences of treating risk as an illness.

## Supporting information

S1 Table Semistructured interview guide in English. (DOCX)

S2 Table Semistructured interview guide in the original language (Spanish). (DOCX)
