# Supplementary material for: When risk becomes illness: The personal and social consequences of cervical intraepithelial neoplasia medical surveillance
Source: PLoS One. 2019 Dec 16;14(12):e0226261. doi: 10.1371/journal.pone.0226261 (PMC6913976; doi:10.1371/journal.pone.0226261)
Supplement: S1 Table — (DOCX) [file pone.0226261.s001.docx]

**S1 Table. Semistructured interview guide in English**

| Experience receiving the diagnosis   - *What do you remember about when you were given the CIN diagnosis?*   *Probes: Who was the health professional who gave you the diagnosis? How did she/he approach it? What were your feelings at that time?* |
| --- |
| Experience living with CIN  Management of information (transmission modes, treatment, lifestyle changes) and information sources:   - *Could you please explain how one acquires an HPV infection? And how could you transmit it?* - *How do you think CIN can be treated?* - *Since the diagnosis, what changes have you made to your lifestyle?* - *How would you describe the information you received during your health care process? How did you resolve your doubts?*   Description of the advice given by health care professionals:   - *How would you describe the advice given by the healthcare professionals who treated you?* - *What was your relationship like?* - *What kind of support did they offer you?*   Personal and social consequences associated with the diagnosis – family, friends, partner, sexuality and motherhood:   - *Have you talked with your family about your diagnosis? How have your relatives reacted? Who has been your main source of support and why?* - *Have you talked with your friends about your diagnosis? How did they react?* - *For participants in a relationship: Have you talked with your partner about the diagnosis? Has your partner sought information about your diagnosis? How would you describe the support your partner has given you? Has your diagnosis resulted in any change in your relationship or in your sexual activities?* - *For participants without a partner: How has your diagnosis influenced your consideration of a new relationship?* - *For participants who did not have children or who wanted to be mothers again: Do you think CIN could affect your pregnancy or fertility? How?* |
| Experience summary   - *Could you summarize how you have felt since you received your diagnosis?* - *We have finished the interview; is there anything else you would like to mention about your experience?* |
