# Supplementary material for: When risk becomes illness: The personal and social consequences of cervical intraepithelial neoplasia medical surveillance
Source: PLoS One. 2019 Dec 16;14(12):e0226261. doi: 10.1371/journal.pone.0226261 (PMC6913976; doi:10.1371/journal.pone.0226261)
Supplement: S2 Table — (DOCX) [file pone.0226261.s002.docx]

**S2 Table. Semistructured interview guide in the original language (Spanish).**

| Experiencias relativas al momento en el que diagnóstico fue comunicado   - *¿Qué recuerdas sobre el momento en el que te comunicaron el diagnóstico?*   *¿Cuál fue el profesional sanitario que te comunicó el diagnóstico?, ¿cómo lo hizo?, ¿qué sentiste en ese momento?* |
| --- |
| Vivir con Neoplasia Intraepitelial Cervical (NIC)  Manejo de información (modos de transmisión, tratamiento, cambios en el estilo de vida) y fuentes de información:   - *¿Podrías explicarme cómo se adquiere la infección por VPH?, ¿y cómo se puede trasmitir?* - *¿Cómo crees que se puede tratar la NIC?* - *Desde que fuiste diagnosticada de NIC, ¿cuáles fueron los cambios que llevaste a cabo en tu estilo de vida?* - *¿Cómo describirías la información que recibiste durante el proceso asistencial?, ¿cómo resolviste tus dudas?*   Descripción sobre el asesoramiento brindado por los profesionales sanitarios:   - *¿Cómo describirías el asesoramiento brindado por parte de los profesionales sanitarios que te atendieron durante el proceso asistencial?* - *¿Cómo fue la relación que se estableció con ellos?* - *¿Qué tipo de apoyo te ofrecieron?*   Consecuencias personales y sociales asociadas al diagnóstico – familia, amigos, pareja, sexualidad y maternidad:   - *¿Has hablado con tus familiares sobre el diagnóstico?, ¿cuál fue su reacción?, ¿quién fue tu principal apoyo?* - *¿Has hablado con tus amigos sobre el diagnóstico?, ¿cuál fue su reacción?* - *Para las participantes que tienen una relación de pareja: ¿has hablado con tu pareja sobre el diagnóstico?, ¿tu pareja ha buscado información acerca del diagnóstico?, ¿cómo describirías el apoyo proporcionado por parte de tu pareja?, ¿tu diagnóstico ha supuesto algún cambio en tu relación de pareja o en la vivencia de la sexualidad?* - *Para las participantes que no tienen una relación de pareja: ¿tu diagnóstico ha influido de alguna manera en plantearte una nueva relación de pareja?* - *Para las participantes que no tienen hijos o quieren volver a ser madres de nuevo: ¿crees que el diagnóstico puede tener efectos en el embarazo o en la fertilidad?, ¿de qué manera?* |
| Resumen de la experiencia vivida   - *¿Me podrías hacer un resumen de cómo te has sentido desde que te comunicaron el diagnóstico?* - *Hemos finalizado la entrevista, ¿quieres hacer alguna aportación más sobre de tu experiencia?* |
